# Disseminated Intravascular Coagulation Due to Drinking Tonic Water

**DOI:** 10.7759/cureus.20512

**Published:** 2021-12-19

**Authors:** Sasmith R Menakuru, Adelina Priscu, Ahmed Salih, Vijaypal Dhillon

**Affiliations:** 1 Internal Medicine, Indiana University Health Ball Memorial Hospital, Muncie, USA

**Keywords:** history taking, hematology disorders, tonic water, quinine, disseminated intravascular coagulation (dic)

## Abstract

Quinine has been used worldwide to treat malaria; however, it is now used as an agent for night-time muscle cramping. The compound, derived from Cinchona tree bark, is found in antimalaria medication, supplements for leg cramping, and beverages such as tonic water and bitter lemon. Quinine, however, is not without its side effect profile which includes a wide range of ailments ranging from nausea to disseminated intravascular coagulation. The authors present a case of a 35-year-old man diagnosed with disseminated intravascular coagulation due to an excessive intake of tonic water because his friend told him that it would help alleviate nighttime leg cramping. We strive to inform physicians about the side effect profile of quinine and stress that a pertinent history must be elicited in patients with unknown causes of disseminated intravascular coagulation.

## Introduction

Quinine is a well-known naturally occurring substance from the bark of Cinchona trees found in parts of western Africa, South America, and the Caribbean islands. Quinine was used as a medication to fight malaria and has been successful; however, there has been a decline in usage as better compounds with fewer side effects have been developed [1]. It is known for its bitter taste and thus has been used in Tonic water since the 1850s. When combined with gin, tonic water has become a staple in bars to form the ubiquitous gin and tonic. The other notable use of quinine in tonic water is for night-time leg cramping; however, the FDA does not recommend this [2]. Quinine is known to have an extensive side effect profile of tinnitus, nausea, vomiting, confusion, acute kidney injury, thrombocytopenia, and most severely disseminated intravascular coagulation [3]. We present a case of a 35-year-old male who presented with disseminated intravascular coagulation with no past medical history, took no medications, and on further examination, was found to be drinking tonic water exclusively for night-time leg cramping for 10 days.

## Case presentation

A 35-year-old male with no prior medical history presented to the emergency department with fatigue, palpitations, bright red blood per rectum, and one episode of hematemesis. Symptoms started overtly about five hours before the presentation, and he was unable to pinpoint what was causing his symptoms. He had no history of trauma, change in medication, and said that he did not change his diet. He said that he occasionally drinks alcohol but has no history of smoking or using illicit drugs. There was also no family history of any hematological disease. On further investigation after calling his wife, it was found that he had been suffering from leg cramps at night and therefore a friend told him that he should drink tonic water. It was made apparent that the patient had been drinking tonic water containing quinine in lieu of water for the past ten days. Examination of the patient revealed a lethargic and hypoxic appearing middle-aged man in mild distress, with a heart rate of 125 beats/min, blood pressure was 110/76 mmHg, respiratory rate of 30/min, and a temperature of 38.3 degrees Celsius. His blood glucose was elevated to 255 mg/dL. He had diffuse abdominal tenderness without rebound tenderness or hepatosplenomegaly, and the rest of the examination was normal. He was subsequently admitted to the Intensive Care Unit after his labs pointed to a picture of disseminated intravascular coagulation. His symptoms worsened over the next three days before he showed improvement.

Investigations

**Figure 1:**
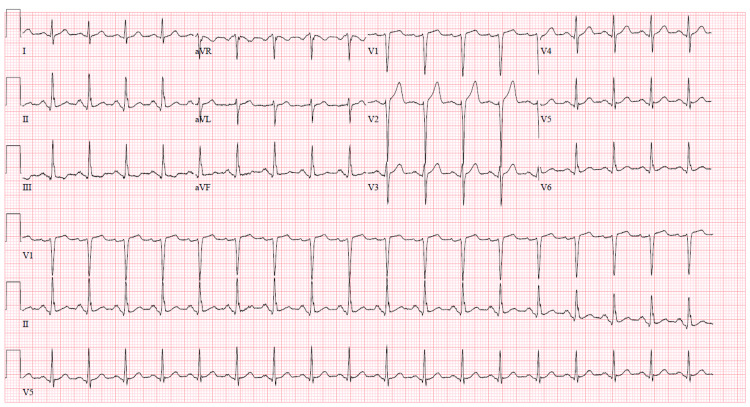
EKG showing sinus tachycardia

Complete blood count with differential, complete metabolic panel, prothrombin time, activated partial thromboplastin time, international normalized ratio, D-dimer, and fibrinogen were ordered (Table 1). An electrocardiogram showed sinus tachycardia but was otherwise normal (Figure 1). The urine toxicology screen was negative, as were a chest x-ray, blood, and urine cultures. His urinalysis showed RBC and hemoglobin in his urine. A peripheral blood smear was routine, and a direct globulin test was positive along with positive quinine-induced platelet autoantibodies, which came back positive two weeks later.

**Table 1:** Laboratory values on admission, day 1, day 2, at discharge, and at 4-week follow up

Laboratory test (reference range)	On admission	Day 1	Day 2	At discharge	At follow-up in 4 weeks
White Blood Cells (4.0–10.8 K/µL)	1.9 K/µL	2.0 K/µL	2.3 K/µL	7.4 K/µL	7.9 L/µL
Hemogloblin (12.0–16.0 g/dL)	6.0 g/dL	7.5 g/dL	8.0 g/dL	13.2 g/dL	13.1 g/dL
Platelets (120–430 K/µL)	11 K/µL	10 K/µL	15 K/µL	154 K/µL	249 K/µL
Neutophils (36–75%)	31%	33%	32%	59%	60%
Lymphocytes (20–50%)	7%	14%	21%	30%	31%
Creatinine (0.5–1.1 mg/dL)	1.9 mg/dL	1.97 mg/dL	1.5 mg/dL	0.72 mg/dL	0.7 mg/dL
Prothrombin Time (12.0-14.7 seconds)	22.1 seconds	25.7 seconds	18 seconds	13.2 seconds	12.9 seconds
Activated Thromboplastin Time (23.3-35.7 seconds)	63.1 seconds	71.4 seconds	60.2 seconds	28.6 seconds	28.9 seconds
International Normalized Ratio (<4 seconds)	1.99 seconds	2.42 seconds	1.52 seconds	1.02 seconds	0.99 seconds
D- Dimer (0-499 ng/mL)	> 10,000 ng/mL	>10,000 ng/mL	4,212 ng/mL	2 ng/mL	-
Fibrinogen (193-473 mg)	57 mg	59 mg	90 mg	342 mg	330 mg
Lactate Dehydrogenase (100-225 µ/L)	700 µ/L	-	-	-	193 µ/L
Reticulocyte Count (0.5-2.0%)	3.32%	-	-	-	-

Differential diagnosis

The primary differential diagnosis were those that caused bleeding, hypercoagulability, thrombocytopenia. We ruled out hemolytic uremic syndrome (HUS), thrombotic thrombocytopenic purpura (TTP), sepsis, induction by drugs or herbal supplements, neoplastic conditions, and autoimmune diseases such as SLE. HUS and TTP were ruled out due to increased PT and aPTT, and no other symptoms were consistent with both conditions. Sepsis was initially the diagnosis made, and the patient was started on empiric vancomycin and piperacillin-tazobactam for broad-spectrum coverage but was later ruled out as none of the investigations pointed to a septic picture. His ANA was negative, and lupus anticoagulant was not detected, and he did not have any hematologic cancer as confirmed by the blood smear. He had no history of neoplastic conditions or family history of bleeding disorders. Since the patient was not taking any medications and tonic water containing quinine was the only change, it was concluded that the tonic water was the cause of his disseminated intravascular coagulation, which was then confirmed by the quinine induced autoantibody test.

Treatment

The patient was admitted to the intensive care unit and was promptly treated with two rounds of blood transfusions with packed red cells, given his hemoglobin was 6 g/dL. Repeat hemoglobin the next day was 7.5 g/dL, and he was given one more unit of packed red cells. He was otherwise treated conservatively with supportive care with his hemoglobin, platelet count, coagulation profile, basic metabolic panel, and electrolytes monitored daily. Tonic water was immediately stopped, and he was educated about the side-effect profile of quinine. He responded well after the first day to intravenous fluids and electrolyte placement. Broad-spectrum antibiotics were stopped after negative blood and urine cultures came back. His coagulation profile, hemoglobin, and platelets normalized after six days. He followed up with the hematologic clinic five days after discharge and again in four weeks, and both times his labs were within his normal limits (Table 1).

## Discussion

Quinine, a naturally occurring substance from the bark of the Cinchona tree, is currently used as an antimalarial medication, a supplement for leg cramping, and a flavoring agent in beverages such as tonic water bitter lemon. In the United States, the FDA limits the contents of quinine in Tonic Water to 83 mg/L, as the therapeutic effect ranges from 500 to 1,000 mg [4]. Commercially produced tonic water contains carbonated water, sugars either natural or high fructose corn syrup, quinine, natural flavors, and preservatives (Table 2). It has been widely used in bars around the world and as a home remedy for centuries; however, it is not without its side effect profile. According to the Federal Drug Administration Adverse Events reporting system, 71 cases of adverse effects due to quinine have been reported in 2021, and a total of 1,743 cases since the 1970s. There have been reports of quinine causing drug-induced thrombocytopenia, neutropenia, anemia, acute renal failure, QT prolongation, liver toxicity, neurological abnormalities, and disseminated intravascular coagulation [5]. Currently, the FDA only approves quinine as a treatment for malaria due to the severe side effect profile, but it is widely sold as a supplement for leg cramping and in tonic water [6]. Due to this, quinine usage is often self-regulated, further complicating cases, as patients may not inform physicians about usage, hindering a prompt diagnosis. Our patient drank only tonic water for 10 days, in lieu of water, and thus, we report the only case of our knowledge of a patient drinking tonic water resulting in the developing disseminated intravascular coagulation.

**Table 2:** Nutrition facts of tonic water our patient was consuming

Nutrition facts of 1 bottle of tonic water (1 L)	Amount per bottle	Daily value
Calories	380	--
Total Fat	0g	0%
Sodium	140mg	6%
Total Carbohydrates	100g	38%
Total Sugars	99g	--
-- Includes Added Sugars	99g	198%
Protein	0g	0%
Ingredients: Carbonated Water, High Fructose Corn Syrup, Citric Acid, Sodium Benzoate (Preservative), Quinine, Natural Flavors		

Quinine is a common cause of drug-induced thrombocytopenia and the most common cause of drug-induced microangiopathy [7]. In a systematic review done by Liles et al. of 102 patients from 124 articles, quinine was described as causing an acute immune-mediated reaction. Quinine pills caused 102 reactions (72%), 28 (20%) were caused by quinine-containing beverages, and 12 (8%) by five other types of exposure. Forty-one patients were excluded as they only had dermatologic reactions, 92 out of 101 required hospitalization for severe illness, with 30 requiring renal replacement therapy, and three passed away. The authors of the review concluded that quinine, even only with minimal exposure from common beverages such as tonic water, can cause severe adverse reactions involving multiple organ systems [8].

According to Mandal et al., quinine appears to decrease the excitability of the motor endplate, which reduces the contractility of muscles [9]. Connellan et al. concluded that quinine induced a widespread conformational change in platelet membrane antigen, which subsequently exposes neoantigens leading to the formation of quinine-associated antibodies [10]. Many trials have been done that have proven the effectiveness of quinine on leg cramping [11,12]. Quinine is a prescription medication in the United States for malaria and is currently not approved for the treatment of leg cramps; however, there have been many case reports of patients suffering from side effects of quinine, including disseminated intravascular coagulation by taking tablets from other countries [13]. In certain patients who are susceptible to the effects of quinine, the immune response elicited may result in not only the formation of anti-platelet antibodies but also antibodies against erythrocytes, leukocytes, and endothelial cells. Depending on individual patients, there may be a spectrum of diseases ranging from mild, transient thrombocytopenia to overt DIC [14]. The onset is often abrupt and the time it takes for symptomology to develop is variable, but after quinine cessation, recovery of platelet counts often occurs in seven to 10 days [15].

## Conclusions

We hope this case report brings awareness of quinine as a non-approved use for nocturnal muscle cramping and the side effect profile associated with it. The authors urge physicians to recognize quinine as a possible cause of DIC as prompt treatment offers an excellent prognosis. The importance of eliciting a proper history in patients with unknown DIC cannot be stressed as frequently; the intake of quinine-containing compounds, especially tonic water, are emitted as patients perceive them as safe. We conclude tonic water should be used in moderation, if at all, and the FDA should issue labels on bottles of tonic water containing quinine regarding the side effects of excessive consumption.
